# Eco-Friendly Recovery of Homogalacturonan-Rich Pectin from Flaxseed Cake via NADES Extraction

**DOI:** 10.3390/polym17182532

**Published:** 2025-09-19

**Authors:** Aleksandra Mazurek-Hołys, Ewa Górska, Marta Tsirigotis-Maniecka, Maria Zoumpanioti, Roman Bleha, Izabela Pawlaczyk-Graja

**Affiliations:** 1 Department of Engineering and Technology of Chemical Processes, Faculty of Chemistry, Wroclaw University of Science and Technology, Wyb. Wyspianskiego 29, 50-370 Wroclaw, Poland; aleksandra.mazurek@pwr.edu.pl (A.M.-H.); ewa.gorska@pwr.edu.pl (E.G.); marta.tsirigotis@pwr.edu.pl (M.T.-M.); 2 Institute of Chemical Biology, National Hellenic Research Foundation (NHRF), 48 Vassileos Constantinou Ave., 11635 Athens, Greece; mariaz@eie.gr; 3 Department of Carbohydrates and Cereals, Faculty of Food and Biochemical Technology, University of Chemistry and Technology in Prague, Technická 1905/5, 160 00 Prague, Czech Republic; roman.bleha@vscht.cz

**Keywords:** flaxseed cake, natural deep eutectic solvents, pectin, homogalacturonan

## Abstract

Flaxseed polysaccharides (FLP) are bioactive macromolecules with valuable functional properties and applications in the food, pharmaceutical, and packaging industries. This study focused on obtaining high-purity pectin from flaxseed cake using sustainable extraction with natural deep eutectic solvents (NADES) based on choline chloride (ChCl) and citric acid (CA) The ChCl/CA system (1:1) resulted in the LU3 extract, which provided the best outcome, yielding the highest pectin recovery (36.88 mg/g), elevated uronic acid content (30.33% of sample; 68.15% of saccharides), and the lowest protein contamination (11.46%), confirming superior pectin purity. Structural (UV-Vis, FT-IR, GC-MS, GPC, LH-20) identified homogalacturonan with xylogalacturonan domains (53% DM) and a molecular weight range of 14–500 × 10^3^ g/mol. Morphological and physicochemical characterization, including SEM/EDS imaging, zeta potential analysis, and rheological measurements, revealed that LU3 is an anionic, heterogeneous biopolymer exhibiting pH-dependent charge behavior. These properties underscore its potential as a safe and effective material for bio-industrial applications. Overall, the study demonstrates that NADES provide an eco-friendly and efficient medium for extracting high-quality pectin from flaxseed cake, offering a sustainable strategy for the valorization of flaxseed polysaccharides in bio-based products.

## 1. Introduction

Flax (*Linum usitatissimum* L.) is an annual herbaceous plant that belongs to the genus Linum and family Linaceae. Flax cultivation dates back to more than 7000 BC when flax fiber was used by ancient Egyptians for wrapping mummies and flax oil was used for body preservation [1]. Currently, *Linum usitatissimum* is cultivated mainly for flaxseed oil production. The remaining above-ground parts of the plant are also collected for flax fiber processing. Europe is the largest global producer, meeting as much as 95% of the market demand [2]. Flax fibers are used for textiles, blotting papers, banknotes, polymers of lactic acid biodegradable composite materials, and glass fiber substitutes [3]. According to the FAO (2021), the major flaxseed oil producers are China (32.3%), Belgium (17.4%), and the USA (10.0%) [2], generating large amounts of by-products, also known as ‘cake’ or ‘meal’ [4,5].

According to the FAO (2021) [2], the major flaxseed oil producers are China (32.3%), Belgium (17.4%), and the USA (10.0%), generating large amounts of by-products.

Defatted flaxseed cake is rich in many components that contain bioactive components with valuable functionality, including polyphenols, proteins, and FLP [6,7]. Due to its ben-eficial properties, flax cake is used not only as animal feed for farm animals, i.e., cattle, pigs, poultry, and pets, but also as a food ingredient as a component in human food, e.g., as nutraceutical ingredient or the component improving texture and sensory properties of many daily consumed food products, including sourdough bread, margarine or meat products [1,8]. FLP is used as a gelling agent in the food industry [9]. Moreover, they can stabilize emulsions, e.g., in ice creams and in yogurt [10,11,12]. FLP is also utilized as a fat replacer and a prebiotic in food products such as cream cheese [13,14]. FLP are proven to serve as useful components of food packaging materials and edible coatings [15,16,17,18].

Flax seeds are a rich source of many bioactive compounds, e.g., α-linolenic acid, secoisolariciresinol diglucoside, and soluble as well as insoluble fiber. These compounds are anti-inflammatory, antioxidant, and lipid-regulating agents, supporting therapies against cardiovascular diseases, diabetes, and cancers [19]. The insoluble type of linseed fiber is mainly composed of cellulose, hemicellulose, and lignans, whereas the water-soluble type of this fiber forms linseed mucus [20,21]. The first type of fiber is responsible in the body for facilitating the transport of fecal masses [20] and for the production of the diglucoside secoisolariciresinol (SDG), in the digestion tract, which, when transformed into enterolactone, prevents the formation of colon, prostate, intestinal, and lung cancers [20,22]. In turn, flaxseed mucilage isolated in an aqueous environment consists of one neutral fraction and two acidic fractions. The neutral fraction consists of approximately 75% of arabinoxylan (AX), with a (1–4)-D-xylan backbone substituted by arabinose and short D- and L-galactose chains [2,20,21]. The two remaining acidic fractions, which are typical pectins, are polysaccharides with main chains composed of (1-4)-galacturonic acid and (1-2)-L-rhamnose, which are branched with arabinose, galactose, or arabinogalactan I side chains. These polysaccharides can be classified as rhamnogalacturonans type I (RG I) [20]. The neutral arabinoxylans combined with the pectins rich in rhamnose are the raw material for the synthesis of short-chain fatty acids (SCFA), produced in fermentation catalyzed by intestinal bacteria [20,23]. SCFAs, in turn, act as substrates to synthesize the components essential to life processes, like long-chain fatty acids, cholesterol, glutamine, and glutamates [24].

The valorization of waste materials through pectin extraction offers not only an environmentally friendly strategy to obtain biodegradable, biocompatible, and non-toxic biofilms and biomaterials. Pectins can function as matrices and scaffolds that facilitate cell adhesion and proliferation, making them highly suitable for applications in tissue engineering, including tissue repair and regeneration [25]. For example, Lapomarda et al. demonstrated the use of pectin derivatives to construct 3D scaffolds for ear and nose reconstruction [26]. Pectins are also incorporated into composites, such as cellulose, gelatin, collagen, or polylactic acid, to enhance material properties [25,27]. Pectins are also utilized in drug delivery systems in the form of hydrogels, micro- and macroparticles, biofilms, tablets, and microspheres. These systems enable targeted therapies, such as in cancer treatment or oral drug delivery, as pectin degradation occurs selectively via intestinal bacteria expressing pectinolytic enzymes, ensuring release of the active compound specifically in the colon [28]. In the food industry, pectins are widely applied to produce edible, biodegradable, and non-toxic biofilms aimed at prolonging shelf life. As with biomaterials, pectins are often integrated into composites with starch, polylactic acid, or essential oils, improving mechanical strength and imparting antimicrobial or antioxidant properties [29,30,31]. Furthermore, pectins serve as versatile functional agents in food processing, acting as gelling, thickening, emulsifying, and stabilizing components [10].

So far, various methods for the extraction of polysaccharides from flax seeds have been described, including hot water extraction (HWE) [32,33], ultrasound-assisted extraction (UAE) [32,33], microwave-assisted extraction (MAE) [32,33,34], alkali-assisted extraction (AAE) [23], and enzyme-assisted extraction (EAE) [35]. An increase in process temperature resulted in a higher extraction yield, ranging from 4% to 9.4% (*w*/*w*). In each method used, the extracted polysaccharides were accompanied by a protein fraction, whose proportion increased with temperature and reached 15.1%, 15.7%, and 16.8% for HWE, MAE, and AAE, respectively [32]. However, none of the applied extraction methods enabled the isolation of flaxseed polysaccharides containing more than 36% uronic acids in their sugar composition [32,33,34,35]. The high thickening capacity of flaxseed mucilage polysaccharides significantly increases the viscosity of the extraction medium, which poses a major limitation for the processing of flaxseeds and flaxseed cake in aqueous systems [32]. A potential strategy to address this issue is to reduce the solid-to-liquid ratio; however, this approach may be considered suboptimal from a process scale-up perspective.

In the present study, the polysaccharides rich in uronic acids were extracted from flaxseed cake, a by-product of flaxseed oil production. The research primarily focused on the extraction process from this raw material, employing, among other methods, aqueous solutions of natural deep eutectic solvents (NADES). The aim was to evaluate the potential of NADES as green solvents for the efficient extraction of pectins from this food processing waste, in comparison to other extraction methods. Although aqueous solutions of NADES have been previously reported in the scientific literature as effective extraction media for isolating pectins from various plant-based wastes [36,37,38,39], their use for flaxseed cake valorization has not yet been explored, despite the well-documented richness of this raw material in such macromolecules. The existing literature describes only a single attempt to use NADES on flax seeds, specifically those tailored for lipid extraction, in the context of linseed oil recovery [40]. This article specifically presents the influence of the molar ratio of NADES components, i.e., choline chloride and citric acid, on the yield of pectin extraction and the chemical characteristics of these polysaccharides. The polysaccharide product with the highest galacturonic acid content was subjected to spectrometric analyses, including UV-Vis and FT-IR, as well as chromatographic techniques such as GPC, LH-20, and GC-MS. Additionally, the morphology of this product was characterized by scanning electron microscopy (SEM) coupled with energy-dispersive spectroscopy (EDS). The behavior of this product under different pH and ionic strength conditions was evaluated in terms of zeta potential and viscosity.

## 2. Materials and Methods

### 2.1. Plant Material and Reagents

*Linum* L. seed cake was purchased from the pharmaceutical plant Polpharma S.A. (Poland). Moisture content of flaxseed cake was determined using a moisture analyzer (MB27, Ohaus, Nänikon, Switzerland). Briefly, 5 g of the raw material was dried at 70 °C until a constant mass was reached. As the dry mass was 97.03 ± 0.04% we assumed acceptable to omit this parameter from the calculation of the extraction yield. Choline chloride (≥98%), *m*-hydroxybiphenyl (85%), gallic acid monohydrate, Folin-Ciocalteu reagent, and bovine serum albumin (BSA) were purchased from Sigma-Aldrich (Warsaw, Poland). Citric acid monohydrate, methanol, and ammonia solution (25%) were obtained from P.P.H. STANLAB (Lublin, Poland). Phenol, D-(+)-galacturonic acid monohydrate (>97%), and sodium borohydride (≥99%) were acquired from Fluka (Charlotte, NC, USA). Concentrated sulfuric acid (>95%), disodium-tetraborate-10-hydrate (Na_2_B_4_O_7_ × 10 H_2_O), sodium hydroxide, sodium carbonate anhydrous, and sodium tartrate, trifluoroacetic acid (99.5%), pyridine, acetic anhydride, and sodium sulfate were purchased from POCH (Warsaw, Poland). Copper (II) sulfate (VI) pentahydrate and dichloromethane were obtained from Chempur (Piekary Śląskie, Poland). All solvents were of analytical grade.

### 2.2. NADES Preparation

Four natural deep eutectic solvent (NADES) variants with different molar ratios of choline chloride (ChCl) and citric acid (CA) were prepared, including ratios of 1:1, 1:2, 1:3, and 1:4. To obtain 100 g of the NADES mixture, the chemicals were combined in their respective proportions and mixed under reduced pressure, at 70 °C for 15–30 min. The process continued until a homogeneous liquid was formed. Subsequently, immediately before extraction, NADES was dissolved in distilled water at a ratio of 1:9 *w*/*w* to obtain the final extraction medium.

### 2.3. Extraction of Pectins from Flaxseed Cake

The extraction and purification of pectins from flaxseed cake were performed following standardized procedures described previously [41]. The initial step involved suspending 50 g of flaxseed cake in 1000 mL of various extraction media (distilled water, 10% citric acid, or NADES (ChCl/CA) with molar ratios of 1:1, 1:2, 1:3, or 1:4). The mixtures were then subjected to extraction at 100 °C for 4 h. After extraction, each extract was purified according to the protocol previously described [21]; the solutions were cooled to room temperature and solids were removed by filtration. The extract was then condensed under reduced pressure. The alcohol-insoluble residue (AIR) was precipitated by adding excess collected by vacuum filtration and dried, and stirred continuously for 2 h at room temperature. The precipitate was filtered under reduced pressure and eventually dried.

The crude product was dissolved and dialyzed against distilled water using a Spectra/Por^®^ membrane (MWCO: 12–14 kDa, Spectrum Labs, San Francisco, CA, USA) until the conductivity of the external solution equaled that of distilled water. Finally, the purified solution was dried under reduced pressure to obtain the final product. The yield of the extraction was calculated using the formula:

(1) $$Y\;\left[\%\right]=\frac{mass\;of\;product\;after\;extraction\;process\;\left[mg\right]}{mass\;of\;raw\;material\;\left[g\right]}$$

### 2.4. Chemical Characterization

The conductivity and pH of extraction media solutions were measured using a Laboratory pH/Conductivity meter CPC-511 (Elmetron, Zabrze, Poland). The total carbohydrate content in *Linum* L. products was determined using the phenol-sulfuric acid assay, with glucose as the standard at 490 nm [42]. To quantify uronic acids, the *m*-hydroxybiphenyl method was applied at 520 nm, with galacturonic acid used as the reference compound [43]. Phenolic compounds were measured using the Folin-Ciocalteu method, with gallic acid serving as the standard (λ = 765 nm) [44]. Protein content was determined at 750 nm using the Lowry assay, with BSA as the reference compound [45]. Measurements were taken using a Cecil CE 2021 spectrophotometer and a SPECTROstarNano microplate reader (BMG Labtech, Ortenberg, Germany).

### 2.5. Monosaccharides Composition

The polysaccharide sample was hydrolyzed (2M TFA, 120 °C, 5 h), reduced with sodium borohydride, and acetylated by acetic acid anhydride in the presence of pyridine. Thereafter, the sample was extracted three times with dichloromethane and evaporated to dryness under N_2_ to obtain alditol acetates of neutral monosaccharides, following a commonly used protocol [46]. Briefly, prior to the analysis, the sample was dissolved in 500 µL of dichloromethane. The analysis was performed on the Trace GC Ultra device, coupled to an ITQ 700 mass spectrometer, equipped with a quadrupole ion trap detector (Thermo Scientific). The separation of the analyzed sample was carried out in a Restek RTX-2330 column (0.25 mm × 30 m) with helium as the carrier gas, with a constant flow rate of 1 mL/min. The sample injection volume was 1 µL. The injection port was set in splitless mode and heated to a temperature of 260 °C. The ion source in the detector was heated to 300 °C. The analysis temperature program was set as follows: 170–180 °C (1 °C/min), 180–235 °C (3 °C/min), and finally maintaining 235 °C for 10 min.

### 2.6. FT-IR Spectroscopy Analysis

Fourier-transform infrared spectroscopy (FT-IR) was performed using a Nicolet iS20 FT-IR spectrometer (Thermo Fisher Scientific, Waltham, MA, USA) and Omnic 9.0 software. A sample-containing KBr tablet was prepared using a hand press (Pike Technologies, Madison, WI, USA) to perform the measurement. The spectrum was recorded in the mid-infrared region spanning 4000–400 cm^−1^ with a resolution of 4 cm^−1^ and by collecting 64 scans. The raw spectrum was corrected for atmospheric background and baseline, then smoothed using a 10 ppt filter. The positions of the shoulders were detected using a second derivative algorithm.

### 2.7. Homogeneity

The crude extract was prepared for Sephadex LH-20 gel chromatography to assess its homogeneity. The sample was weighed (~33 mg) and completely dissolved in 0.5 mL of 0.1 M NaOH, followed by centrifugation and filtration on a syringe filter (0.45 μm). The column (13 × 300 mm) was packed with resin (~10 g of Sephadex LH-20), which was saturated with the eluent (methanol/0.1 M NaOH, 1:3), and pre-equilibrated with at least 3 bed volumes of eluent. Then, 0.5 mL of the dissolved crude pectin-like extract was loaded onto the column. The mobile phase was methanol/0.1 M NaOH (1:3). The eluates were collected (1 mL/tube). The carbohydrate profile was analyzed using the phenol-sulfuric acid assay [34]. Phenolic and protein compound levels were assessed using the Folin-Ciocalteu and Lowry methods, respectively [44,45].

Gel Permeation Chromatography (GPC) was performed using Sephacryl S-300 HR resin. The column (20 × 1200 mm) was packed with gel and washed with 0.1 M NaOH to remove residual 0.02% sodium azide solution (at least 3 bed volumes). The crude product (~30 mg) was dissolved in 0.1 M NaOH (0.5 mL). Then, 0.5 mL of the dissolved crude pectin-like product was loaded onto the column. The void volume (V_0_) was 80 mL. Eluates were collected in 2 mL fractions using a Gilson FC 203B fraction collector (Gilson, Middleton, WI, USA) at a flow rate of ~0.5 mL/min. The carbohydrate profile analysis was performed using the phenol-sulfuric acid assay [42].

### 2.8. Physicochemical Characterization

#### 2.8.1. Microscopic Evaluation

Immediately before the experiment, LU3 powder was mounted onto double-sided adhesive carbon tape. A 50 μL drop of LU3 solution (10 mg/mL) was applied to double-sided adhesive carbon tape 24 h before the experiment to allow the water to evaporate. The morphology of LU3 was examined using a scanning electron microscope (JSM-6601LV, JEOL, Tokyo, Japan) (operating at 5–20 kV, spot size 30, high vacuum) coupled with an energy-dispersive X-ray spectroscopy (EDS) system (Oxford Aztec Energy, Oxford, UK) for mineral characterization.

#### 2.8.2. Zeta Potential Measurements

The electrophoretic mobility of LU3 particles was measured at 22 °C using a Zetasizer Nano-ZS analyzer (Malvern, UK) (λ_laser_ = 632.8 nm) in capillary zeta cells. LU3 was dissolved in ultrapure water (Milli-Q, Merck Millipore, Darmstadt, Germany) or 0.1–100 mM NaCl (as a supporting electrolyte) to a final concentration of 0.1% *w*/*v*. The pH of the solutions was adjusted to a range of 3–10 using a pH meter (Mettler Toledo, Columbus, OH, USA) with 0.1 M HCl or 0.1 M NaOH. Each sample was equilibrated for 180 s, and measurements were performed in triplicate, each consisting of 30 runs. The surface charge of LU3 was expressed as the zeta potential (ζ) in millivolts (mV).

#### 2.8.3. Static Viscosity Analysis

The viscosity of 0.1% LU3 was measured in a glass beaker at 23 ± 1 °C using a Rotavisc Lo-vi viscometer (IKA, Poznań, Poland) equipped with an SP-6 spindle, following the requirements of the International Standard ISO 2555:2018 [47]. The pH of the solutions was adjusted to a range of 3–10 using a pH meter (06-700ALS, Chemland, Łódź, Poland) with 0.1 M HCl or 0.1 M NaOH. Each sample was analyzed in triplicate for 120 s at 190 rpm.

### 2.9. Statistical Analysis

Statistical evaluation was carried out with Microsoft Office Excel 2021. Data are expressed as the mean ± standard deviation (S.D.). The statistical significance of differences between the treated and the control groups was determined by the Student’s *t*-test, at a significance level of *p* < 0.05.

## 3. Results and Discussion

### 3.1. Crude Polysaccharides from Flaxseed Cake

**Figure 1:**
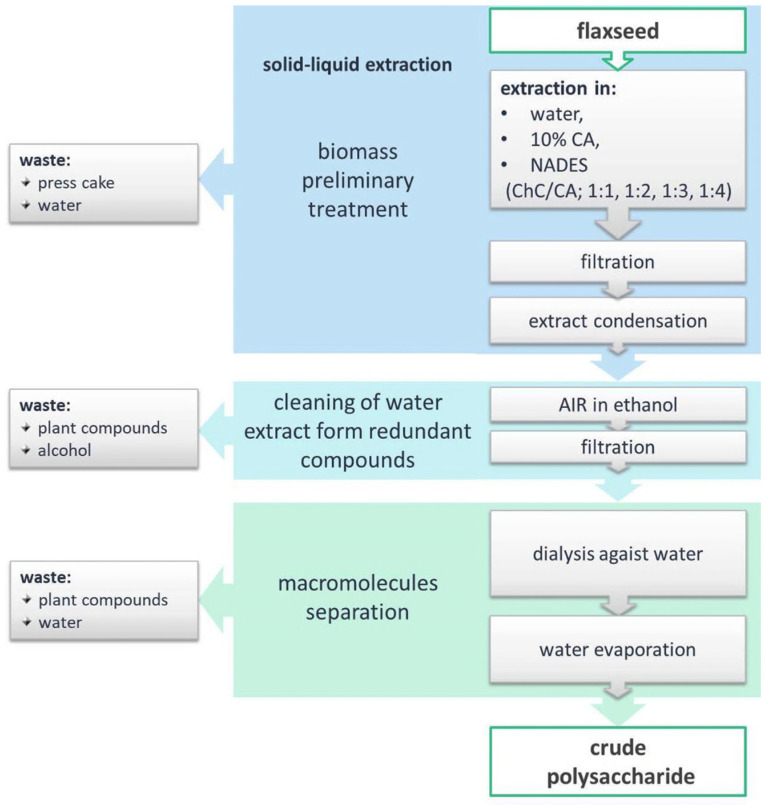
Process of polysaccharides isolation from *Linum usitatissimum* seed cake.

Crude extracts from *Linum usitatissimum* L. seed cake were obtained by hot water extraction (HWE), in a 10% citric acid solution, or using 10% aqueous solutions of natural deep eutectic solvents (NADES) composed of choline chloride (ChCl) and citric acid (CA) in different molar ratios, i.e., 1:1, 1:2, 1:3, or 1:4, all at 100 °C for 4 h. Each crude extract was then processed according to a standard polysaccharide isolation protocol [21]; that is, it was filtered to remove the solid residue, concentrated, and precipitated using methanol to collect the alcohol-insoluble residue (AIR), which was to enrich saccharide content. Finally, crude polysaccharide products were obtained after dialysis against deionized water (Figure 1).

The extraction yield and total saccharide concentration of the crude polysaccharide products from flaxseed cake ranged from 53.58 to 136.71 mg/g and from 34.89% to 45.20% (based on dry flaxseed cake), respectively. The highest extraction efficiency was observed for the LU4 product, obtained using NADES with a ChCl/CA molar ratio of 1:2, whereas the highest saccharide content was found in the LU6 product, corresponding to a ChCl/CA molar ratio of 1:4.

Additionally, LU3 exhibited the lowest level of contamination by proteins, with a value of 11.46%, in the dry crude polysaccharide product. Notably, the most efficient LU3 product was obtained using the extraction medium with the highest conductivity, i.e., 20.3 mS/cm.

However, in this study, alongside the polysaccharide yield, the second most important parameter indicating the presence of pectins was the uronic acid (UA) content. According to Table 1, the extraction medium with a ChCl/CA molar ratio of 1:1 (LU3) yielded the best results in this regard. It exhibited the highest UA content in both the crude product and the saccharide fraction, with values of 30.33% and 68.15%, respectively. The uronic acid content in other samples ranged from 14.35% to 24.64%, which aligns with scientific literature indicating that flaxseed contains 21–36% UA [33]. It is also crucial to assess the amount of pure pectin obtained from the extraction process. This parameter was estimated by considering both the extraction yield and uronic acid content. As presented in Table 1, pectin recovery, calculated per gram of raw material, ranges from 7.72% to 36.88%. The highest value was achieved for the sample extracted in NADES with a ChCl/CA molar ratio of 1:1. These findings provide the rationale for selecting LU3 as the most promising product with respect to chemical characteristics and for subjecting it to further structural analyses.

A high UA content (~68%) in the isolated crude polysaccharide LU3 suggests the presence of pectin. Xyl residues were predominant among the neutral monosaccharide residues in LU3, along with minor amounts of Ara, Gal, and Rha residues, where the Ara content was almost equal to the sum of Gal and Rha. The molar ratio of Xyl to GalA (Table 2) suggests the presence of a combination of homogalacturonan and xylogalacturonan [48] with a degree of methylesterification of 53% in the crude polysaccharide product. Based on the proportion of the sum of GalA and Rha to the sum of Fuc, Ara, Gal, and Xyl [48] (3.13), it can be stated that the xylogalacturonan present in LU3 is typically linear. The very low Rha-to-GalA ratio in LU3 0.045 (Table 1) suggests that rhamnogalacturonan (RG I) is present as a residual component in the flaxseed pectin, as previously observed [49].

These observations are consistent with Safdar et al. (2020), who reported that flaxseed polysaccharides extracted by alkaline-acid extraction (AAE), hot water extraction (HWE), microwave-assisted extraction (MAE), and ultrasonic-assisted extraction (UAE) had comparable monosaccharide compositions, dominated by rhamnose (Rha, ~22%), followed by Glc, Gal, Xyl, Ara, Fuc, and GalA (~7–10%), indicating rhamnogalacturonan-I and xylogalacturonan [32]. Yu et al. (2022) showed that MAE yielded fractions enriched in Rha, Xyl, Ara, and Gal, but with <20% GalA [34], markedly lower than in this study. Flaxseed pectins enriched in neutral monosaccharides show shear-thinning and weak gelling, whereas those dominated by acidic monosaccharides display viscoelastic behavior [50].

Extraction method strongly influences yield and composition. HWE, often combined with pH adjustment or auxiliary treatments, is widely used. Three-stage countercurrent HWE produced flaxseed pectin at 9.8% yield (80 °C, 30 min) [23]. Ding et al. (2014) obtained 3.05% at 70 °C for 4 h, with 78.36% sugar and 4.74% uronic acid (UA) [51]. Microwave-assisted extraction using CA and HCl yielded 5.55% and 11.60%, respectively, consistent with this study [52]. The products contained 72.87% (CA) and 80.99% (HCl) anhydrouronic acid (AUA), and low-methoxyl pectins with a degree of esterification (DE) of ~43% [52].

Safdar et al. (2020) reported yields of 6.44% (AAE), 8.96% (HWE), 7.01% (MAE), and 7.84% (UAE), with carbohydrate contents of 70–84% [32]. Microwave treatment also improved yield: control and 1, 3, and 5 min treatments gave 3.15%, 3.49%, 4.34%, and 4.76%, respectively—still less than half the value in the present work. UA decreased from 17.03% to 11.16% (control: 15.59%), while sugar exceeded 84% [34]. Enzymatic–ultrasonic-assisted extraction achieved >33% yield from defatted flaxseed meal [50]. For MAE and UAE, protein content was low, below LU3 [33,34].

Differences in yield, chemical composition, and DE depend not only on extraction, but also on cultivar and harvest conditions [50]. No prior studies were found on flaxseed pectin isolation using NADES, highlighting a research gap. NADES, especially ChCl/CA, are increasingly studied for pectin extraction. Applications include jackfruit [53], passion fruit [54], *Averrhoa bilimbi* [55], mango [56], kinnow [57], apple [37,58,59], and sweet lime [60]. For example, ChCl/maleic acid (1:1) with microwave support extracted jackfruit pectin (>33% yield, 68% GalA, DE 34.96%) [53]. Pereira et al. (2024) used Subcritical Water Extraction (SWE) and Pressurized NADES (P-NaDES; ChCl/Glc/water, 1:1:3) for passion fruit, achieving 19.1–27.6% yield (GalA 68%, DE > 50%), while SWE produced the highest GalA (78%) but DE < 50% [54]. Shafie et al. (2019) optimized NADES (ChCl/CA, 1:1) for *Averrhoa bilimbi*, obtaining 14.4% yield, GalA-rich pectin with DE 54% [55]. Santra et al. (2023) tested ten ChCl-based NADES; ChCl:maltose (5:2, 70 °C, 4.5 h) gave the best results (35.66% yield, 78.22% GalA, DE <50%) [57].

Vargas et al. (2025) extracted apple pectins using CA and NADES (CA/Glc/water; lactic acid/Glc/water), yielding 2.5–12.2%, with >76% carbohydrates, UA > 50%, and DE > 50% [37]. NADES were also used to pretreat apple pomace before HWE (ChCl/glycerol, ChCl/lactic acid, potassium carbonate/glycerol, ChCl/oxalic acid, ChCl/urea) [58,59]. Rai et al. (2025) applied ultrasonic cavitation with ChCl/CA, yielding lime pectin at 37.21% with DE 85.49% [60]. Beyond pectins, NADES (e.g., ChCl/CA, 1:1) have also been applied to extract phenolic compounds from olive pomace and black carrot [61].

**Figure 2:**
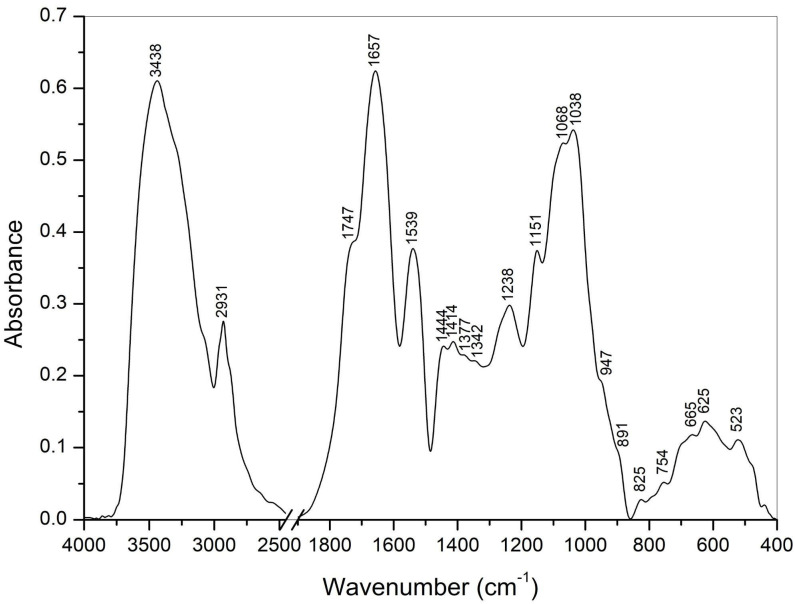
FT-IR spectra for LU3 crude polysaccharides.

Figure 2 presents the FT-IR spectrum of the LU3 product. A broad, intense band around 3438 cm^−1^ corresponds to O-H stretching vibrations, indicating the presence of water, polyhydroxy compounds such as carbohydrates, and polyphenols [62,63]. A sharp, less intense band at 2931 cm^−1^ corresponds to C-H stretching vibrations in CH_3_, CH_2_, and C-H moieties. The shoulder around 1747 cm^−1^ arises from C=O stretching in carboxylic acids and ester groups [62].

Two intense bands centered at 1656 and 1538 cm^−1^, corresponding to amide I and amide II vibrations, respectively, confirm the presence of proteins [64]. This result is consistent with the colorimetric assay, which indicated a protein content of approximately 11.5% in the sample. Two weak bands at 1444 and 1377 cm^−1^ are associated with the anti-symmetric and symmetric bending vibrations of CH_3_ groups [63]. The band at 1414 cm^−1^ indicates symmetric stretching of COO^−^ in salts of uronic acids [62,63].

The band at 1238 cm^−1^, with a high-wavenumber shoulder, is attributed to C-O and C-O-C stretching vibrations in carboxylic acids and ester groups, as well as amide III vibrations in proteins. The narrow band at 1151 cm^−1^ corresponds to the C-O-C stretching vibration of glycosidic bonds. Strong overlapping bands at 1068 and 1038 cm^−1^ originate from C-O and C-C stretching vibrations in pyranoid rings, characteristic of plant cell-wall polysaccharides [65]. The shoulder at 947 cm^−1^ corresponds to C-O bending vibrations, characteristic of pectin structures [66]. The weak band at approximately 825 cm^−1^ and a shoulder near 891 cm^−1^ indicate α- and β-anomeric configurations in carbohydrate units [67]. The bands observed at 754, 665, 625, and 523 cm^−1^ primarily arise from complex skeletal vibrations in the pyranoid ring and amino acid side chains, and to a lesser extent, from low-frequency vibrations in the carboxylic and amide groups of carbohydrates and proteins [68,69,70].

### 3.2. Homogeneity of LU3

**Figure 3:**
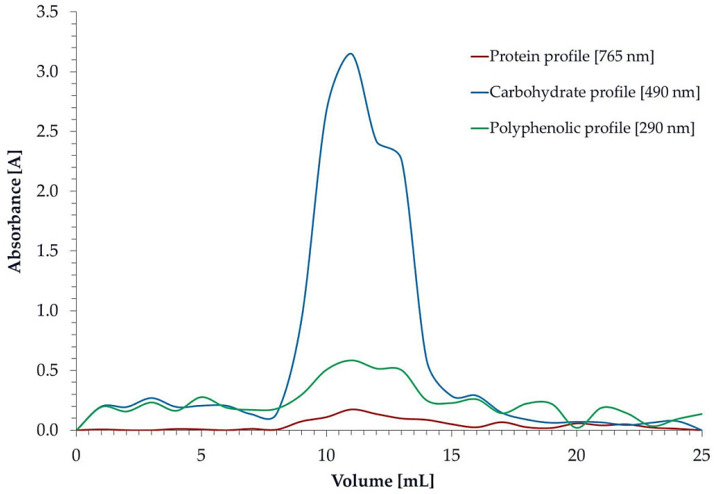
Sephadex LH-20 chromatography of LU3 crude polysaccharide.

The flaxseed cake polysaccharide product LU3, obtained by extraction in NADES (ChCl/CA, 1:1), was separated chromatographically on the lipophilic resin Sephadex LH-20. The collected samples were analyzed for carbohydrates, polyphenols, and proteins [42,44,45]. Fractionation revealed high homogeneity in the analyzed LU3 product, with two main overlapping fractions (Figure 3).

Similar patterns were observed in the saccharide and polyphenolic profiles, although the peaks in the latter were less intense. Additional peaks in the polyphenolic profile were detected at elution volumes of approximately 18 and 21 mL. The protein profile exhibited a broad, flat peak corresponding to the carbohydrate and polyphenolic profiles. These results suggest the presence of polyphenol-polysaccharide conjugates in LU3.

**Figure 4:**
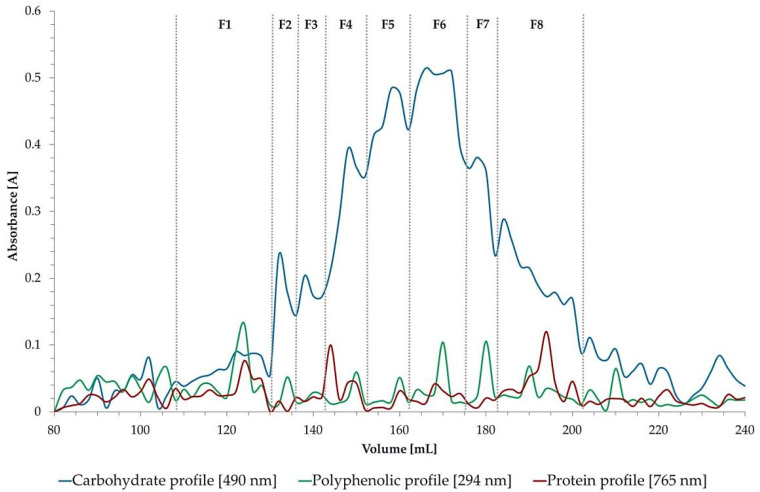
Gel permeation chromatography of LU3 on Sephacryl S300 HR column. Estimated molecular masses are expressed as g/mol.

Gel permeation chromatography (GPC) was performed to determine the molecular size distribution of the LU3 sample (Figure 4). The Sephacryl S300 HR column revealed the complex and molecularly heterogeneous nature of the analyzed sample. A certain degree of heterogeneity was noticeable. The correlation between carbohydrate, polyphenolic, and protein profiles was not clearly visible; however, the carbohydrate and polyphenolic profiles enabled the identification of eight fractions. The number of polyphenolic compounds and proteins in the LU3 sample was quite low, as can be clearly seen in the profile. The average molecular mass of the fractions ranged from approximately Mp ~500 × 10^3^ g/mol to ~14 × 10^3^ g/mol.

The literature reports varying data on the molecular weight of polysaccharides isolated from linseed, mainly because the extraction type and method strongly influence the final product. In general, flaxseed polysaccharides are heteropolysaccharides, composed of homogalacturonan, arabinogalactans, and rhamnogalacturonan, which makes their structure quite complex [71]. The molecular weight of polysaccharides is related to their solubility—lower molecular weight corresponds to higher solubility [72]. Guo et al. (2017) characterized pectic polysaccharides extracted from flaxseed mucilage and kernel as rhamnogalacturonan-I (RG-I), with molecular weights ranging from 285 kDa (mucilage) to 550 kDa (kernel) [73]. A previous study by Goh et al. (2006) identified three molecular weight fractions in flaxseed polysaccharides: 100, 67, and 31 kDa [74]. Safdar et al. (2020) examined flaxseed polysaccharides isolated via AAE, HWE, UAE, and MAE, and found substantial heterogeneity, with products comprising at least two fractions with molecular weights ranging from 5.12 × 10^2^ to 1.33 × 10^6^ g/mol [32].

Based on a literature review, Fabre et al. (2015) determined that flaxseed pectins may correspond to chromatographic peaks between 1.7 × 10^4^ to 1.5 × 10^6^, whereas neutral arabinoxylans exhibit peaks between 1.2 × 10^6^ and 1.5 × 10^6^ g/mol, which may further contribute to variations in viscosity [33].

The differences in the molecular weight of pectins appear to depend largely on the extraction or purification method used.

### 3.3. Physicochemical Properties of LU3

#### 3.3.1. Morphology of LU3

**Figure 5:**
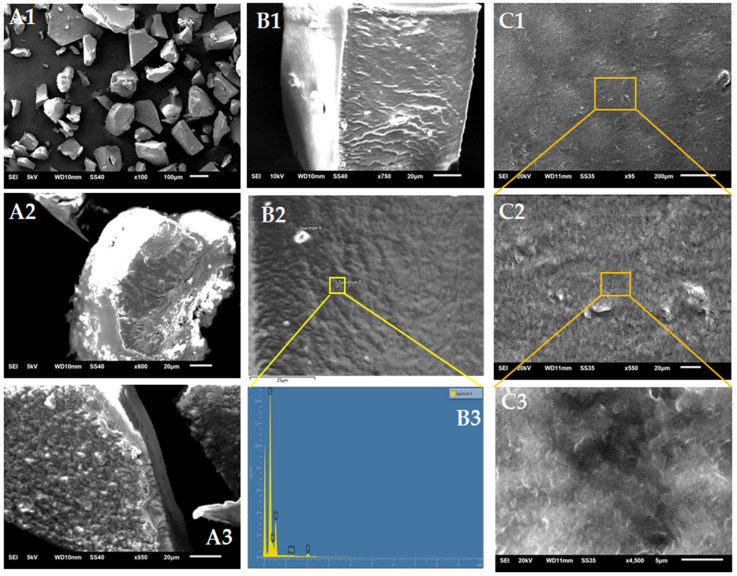
SEM images of surface: LU3 particles (**A1**–**A3**) and LU3 film (**C1**–**C3**) (orange lines indicate region observed with higher a magnification). SEM-EDS images of the surface and mineral characteristics of the LU3 particle (**B1**–**B3**) (yellow lines indicate region mapped for EDS).

Structural features of pectins are important for understanding the physical stability and performance of pectin-based materials, such as gels, films, and encapsulation matrices. Moreover, the morphological changes observed under different environmental conditions can provide useful insights for optimizing pectin functionality in systems where texture, surface properties, and integrity are critical, including controlled release carriers, edible coatings, and hydrogel scaffolds. SEM analysis allowed a detailed visualization of LU3 microstructure at high resolution, showing surface morphology, porosity, and the presence of fibrillar or particulate features of LU3 powder (Figure 5A) and the dry film of LU3 (Figure 5C).

LU3 particles had an irregular shape, with sizes ranging from 36 to 216 µm. The surface of the particles appears coarse, with shallow wrinkles and a few lumps. Similar particle morphology has been reported for pectins derived from eggplant waste [75] and pistachio green hulls [76]. EDS mineral analysis of the LU3 pectin surface detected, in addition to C (62.1 wt%) and O (26.8 wt%), small amounts of N (9.7 wt%) and trace amounts of P (1.1 wt%), Mg (0.2 wt%), and S (0.1 wt%) (Figure 5B). The noticeable nitrogen content suggests the presence of protein complexed with LU3 pectin. The pectin film surface of the LU3 is compact, showing only slight corrugations and no visible pores. At 4500× magnification, fiber-like structures were visible on the surface of the LU3 film, likely cellulose residues from the linseed cell wall [77]. The morphology of LU3 particles seems to be mainly influenced by the randomness of pectin flocculation during the drying process after dialysis, whereas in the film, the LU3 chains become more organized as water gradually evaporates from the surface.

#### 3.3.2. Zeta Potential Profiles of LU3

Zeta potential analysis provides quantitative information on the surface charge and colloidal stability of pectin dispersions across different pH values and ionic strengths. High absolute ζ values (typically > |30| mV) are indicative of stable dispersions with strong electrostatic repulsion, reducing the risk of aggregation or flocculation. The pH-dependent zeta potential profiles for polyanions such as pectins reflect the degree of ionization of carboxyl groups, which is key for understanding interactions with ions, proteins, and other compounds. The influence of ionic strength on ζ has direct implications for pectin behavior in physiological, food, or formulation-relevant environments.

**Figure 6:**
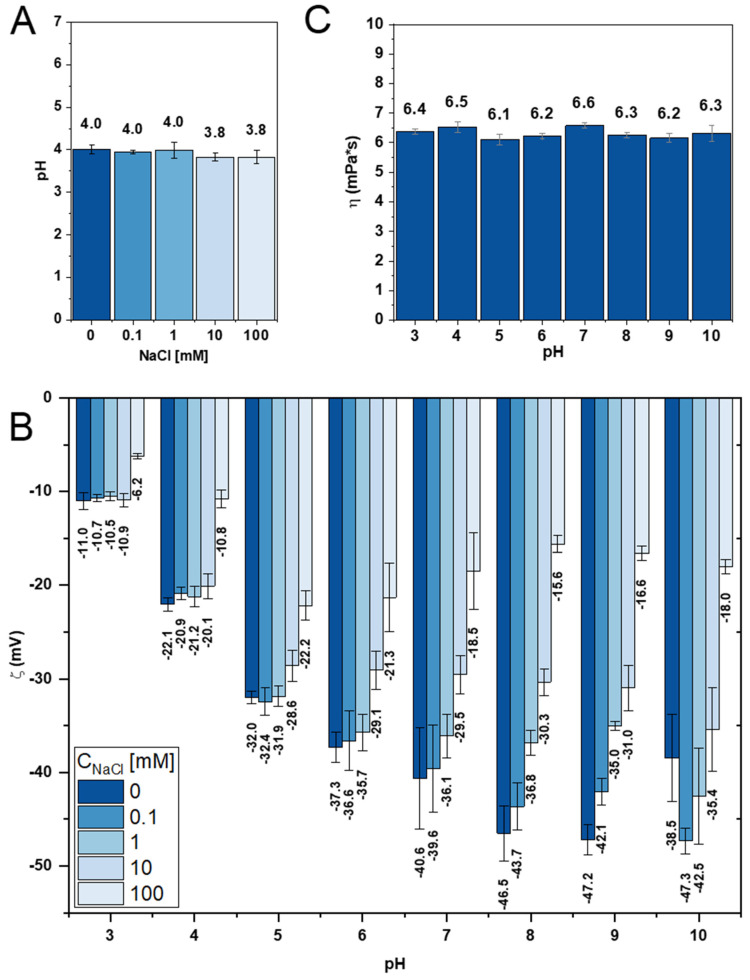
Physicochemical properties of LU3: pH dependence on ionic strength (**A**); zeta potential (ζ) dependence on ionic strength (**B**); viscosity (η) dependence on pH measured in deionized water (**C**).

Overall, a negative correlation was observed between LU3 surface charge and solution pH: as the pH increased, the zeta potential (ζ) decreased from ζ = –11.0 mV at pH 3.0 to ζ = –47.2 mV at pH 9.0. A similar trend was observed for LU3 dissolved in NaCl solutions, regardless of ionic strength, except that the lowest zeta potential was recorded at pH 10. This indicates that the deprotonation of carboxyl residues in GalA units within LU3 increases as pH rises above the pKa of the carboxyl group (~3.5) [78], leading to a higher density of negative charges along the polysaccharide chain. However, changes in LU3 charge density were not linear with pH but varied across the pH range (Figure 6C), which is typical for polyelectrolytes. The steepest decrease in ζ s occurred between pH 3.0 and 5.0, regardless of ionic strength. Under these conditions, protonation of carboxyl groups is most prominent, resulting in substantial neutralization of negative charges in acidic media.

The effect of ionic strength on LU3’s zeta potential further supports these findings. As ionic strength increases, the ζ becomes less negative, consistent with the electrochemical double-layer compression phenomenon: salt ions screen negative surface charges and reduce electrostatic repulsion. However, under acidic conditions, this effect diminishes, showing that protonation of carboxyl groups dominated over counterion interactions in determining surface charge. Compared with pH, ionic strength had a smaller effect on the charge density of LU3 than pH. At pH 3–4, similar ζ values were observed across solutions with low to moderate ionic strength (0–10 mM NaCl). At pH 5–6, this similarity was observed only at lower ionic strengths (0–1 mM). As ionic strength increased, the variation in ζ with pH diminished, suggesting that sodium counterions increasingly screened the negative charges on LU3 by sodium counterions.

The average surface electric charge of LU3 was determined using dynamic light scattering combined with the microelectrophoresis technique and expressed as the zeta potential (ζ). The ζ values represent the sum of the individual surface charges of different functional groups in the biopolymer. The surface charge of LU3 was measured as a function of pH (3.0–10.0) and ionic strength (0.1–100 mM NaCl) (Figure 6B) to assess their combined effect of pH and ionic strength on the zeta potential.

To ensure clarity, the effect of ionic strength on the pH of LU3 was first examined: pH = 4.0 in water, 0.1 mM, and 10 mM NaCl; pH = 3.8 in 10 mM and 100 mM NaCl (Figure 6A). The ζ potential of LU3 in its native form, i.e., pectin dissolved in distilled water (pH ~ 4.0), was −22.1 mV, confirming its anionic character. At pH 7, ζ = −40.6 mV, consistent with observations for apple waste pectin extracted with citric acid [79].

A similar trend in zeta potential behavior has been reported for banana passionfruit epicarp polysaccharides [78]. Furthermore, the degree of deprotonation decreased with increasing ionic strength, reaching a plateau in 100 mM NaCl at pH 7.0. Only minor deviations in ζ were observed within the measurement error, indicating that LU3 had reached an electrostatic saturation point. At this stage, the polymer was saturated with counterions, and the surface charge density could be considered stable. The standard deviations in ζ values were higher at alkaline pH, suggesting that LU3’s fibrillar structure becomes less stable under these conditions.

Similar behavior has been observed for citrus polysaccharides [80]. The optimal ζ potential for proper dispersion of LU3 in aqueous media (ζ < −30 mV) was achieved at pH > 5.0 in water or in solutions of low ionic strength, indicating good colloidal stability. In contrast, in high-ionic-strength solutions and/or at pH < 5.0, the reduced ζ potential suggests that LU3 may fold into semi-helical structures, aggregate, or even flocculate due to weakened interchain repulsion [81]. No complete neutralization of its carboxyl groups by counterions was detected, and LU3 did not acquire a positive charge under any of the tested conditions. These results also suggest that the protein fraction complexed with LU3 is negligible, as it does not significantly affect the net surface charge. In summary, these findings support the classification of LU3 as an unchanged but smoothed commas and parallelism.

#### 3.3.3. Static Viscosity Characteristic of LU3

LU3 is an example of a high-molecular-weight, high-methoxy pectin (HMP), and both of its structural properties contribute to the rigidity of the pectin chain, which corresponds to its higher viscosity [82]. The results show that the apparent viscosity of LU3 remained relatively stable, with only minor variations, regardless of pH (Figure 6C). The highest viscosity values for LU3 were observed in its native state (pH ~4.0) and at pH 7.0, measuring 6.5 mPa·s and 6.6 mPa·s, respectively. Under acidic conditions, both below and above the pKa of carboxyl groups (pKa_COOH), viscosity decreased slightly to 6.2 ± 0.1 mPa·s, while under alkaline conditions, it was approximately 6.3 ± 0.1 mPa·s. These values are comparable to those reported for HMP extracted from jackfruit seeds [83]. No clear correlation was observed between viscosity, pH, or zeta potential. Nevertheless, it is noteworthy that, despite the very low concentration of the LU3 working solution (only 0.1%), its viscosity remains noticeably higher than that of water. Viscosity is a crucial parameter in industrial applications, not only for optimizing extraction but also for predicting functional properties in final products, such as thickening or stabilizing effects [84].

The results of this study suggest that LU3 is a promising candidate for use as a biomaterial in both the food and pharmaceutical industries. Its high galacturonic acid (GalA) content (>68%) contributes to a stable and uniform charge distribution along the pectin chain, enhancing its hydrophilic properties. Simultaneously, the low degree of methylation (DM) and minimal polyphenol content result in only a few hydrophobic regions. Moreover, the well-characterized structure of LU3 with pH-dependent charge behavior allows for predictable chemical and physical interactions with various materials. Therefore, further studies will focus on evaluating LU3 for its repulsive or attractive interactions with other biopolymers, ability to form ionic complexes, water retention and wetting properties, as well as rheological properties such as gelation capacity and viscosity modification, plasticizing effect, barrier properties, and ability to modify the release profile of different formulations.

## 4. Conclusions

In this study, linseed cake polysaccharides were obtained with a focus on sustainable development. Extractions were performed using NADES solutions based on choline chloride and citric acid in various molar ratios. Particular attention was paid to obtaining a pectin-rich macromolecular product while adhering to the principles of sustainable development. Among the tested samples, the LU3 product was the most promising, showing the highest uronic acid content and the greatest proportion of uronic acids within the saccharide fraction. The NADES with a choline chloride/citric acid (ChCl/CA) molar ratio of 1:1 resulted in the highest pectin recovery and the lowest contamination, yielding a high-purity product. The high conductivity of this extraction medium correlated with its superior extraction efficiency. The most promising LU3 product underwent several spectrometric (UV-Vis, FT-IR) and chromatographic (GPC, LH-20, GC-MS) analyses.

Additionally, the morphology of this product was characterized by scanning electron microscopy (SEM) with energy-dispersive spectroscopy (EDS). The behavior of this product under different pH and ionic strength conditions was assessed via zeta potential and viscosity measurements, which indicated typical polyelectrolyte behavior, confirming its anionic nature and potential to form stable colloidal systems. Owing to its well-characterized chemical properties, LU3 can be utilized as a novel material alongside commonly used polysaccharides such as cellulose, sodium alginate, or chitosan, as well as their chemically modified derivatives. It can serve as a plasticizer, humectant, or building block for local or systemic drug delivery systems, and can also be used in environmentally friendly food packaging to extend shelf life.

Based on these findings, it can be concluded that NADES-assisted extraction appears to be a promising technology for recovering valuable biopolymers from oil industry by-products, aligning with current trends in the circular economy and sustainable development. Carefully selected NADES extraction conditions yield pectin suitable for specific applications, including emulsion stabilization, drug delivery, tissue engineering, and functional food development.

Future studies will focus on isolating pure pectic fractions for detailed chemical analysis. For the fraction exhibiting the most promising characteristics, a structural proposal will be developed based on GC-MS analysis to determine monosaccharide composition, and NMR spectroscopy will provide insights into the linkages between saccharide subunits.

## Figures and Tables

**Table 1:** Colorimetric analysis of samples LU1-LU6 and conductivity and pH of extraction media solutions.

Name of the Samples	Extraction Medium	Conductivity of Extraction Medium (mS/cm)	pH of Extraction Medium	Yield of Extraction (mg/g)	Saccharides (wt%)	Uronic Acids (wt%)	Uronic Acids in Saccharide Part (wt%)	Uronic Acids in Raw Material (mg/g)	Polyphenols (µM GAE/1 g of Dry Product)	Proteins (wt%)
LU1	H_2_O	0.0064	5.22	53.58	34.89 ± 1.36	14.35 ± 0.16	41.13	7.72	94.24 ± 10.80	14.52 ± 0.72
LU2	0:1 *	4.70	2.88	117.21	41.29 ± 2.20	24.41 ± 0.21	59.12	28.61	144.88 ± 3.03	12.00 ± 049
LU3	1:1 *	20.30	2.94	121.62	40.14 ± 1.92	30.33 ± 2.39	68.15	36.88	174.56 ± 11.35	11.46 ± 0.41
LU4	1:2 *	15.63	2.90	136.71	39.45 ± 1.56	24.64 ± 0.58	61.44	33.71	200.69 ± 16.02	12.18 ± 0.28
LU5	1:3 *	13.38	2.89	133.43	38.60 ± 0.75	23.15 ± 0.30	59.97	30.91	194.16 ± 16.77	13.01 ± 0.32
LU6	1:4 *	11.44	2.88	119.26	45.20 ± 1.21	24.37 ± 0.25	53.92	29.06	182.88 ± 8.52	11.95 ± 0.31

* Molar ratios of NADES components: choline chloride/citric acid. The extraction was performed in 10% (*m*/*v*) solutions of NADES.

**Table 2:** Monosaccharide composition and degree of esterification of LU3.

	Monosaccharides Composition (wt%)							DM ^b^ (%)
Fuc	Rha	Ara	Xyl	Man	Gal	Glc	GalA ^a^	
0.12 ± 0.01	3.06 ± 0.15	7.67 ± 0.38	11.90 ± 0.60	1.09 ± 0.05	3.08 ± 0.15	0.77 ± 0.24	68.31 ± 3.42	53

^a^ GalA—galacturonic acid content (wt%) calculated based on *m*-hydroxybiphenyl assay, as described by Blumenkrantz and Asboe-Hansen (1973) [43]. ^b^ DM—degree of methylesterification of carboxylic functional groups of GalA, based on the FT-IR spectrum.

## Data Availability

The original contributions presented in this study are included in the article. Further inquiries can be directed to the corresponding authors.
